# A Fiber Optic Catalytic Sensor for Neutral Atom Measurements in Oxygen Plasma

**DOI:** 10.3390/s120403857

**Published:** 2012-03-26

**Authors:** Rok Zaplotnik, Alenka Vesel, Miran Mozetic

**Affiliations:** 1 Jozef Stefan Institute, Jamova 39, Ljubljana 1000, Slovenia; 2 Center of Excellence for Polymer Materials and Technologies, Tehnoloski Park 24, Ljubljana 1000, Slovenia; E-Mails: alenka.vesel@ijs.si (A.V.); miran.mozetic@ijs.si (M.M.)

**Keywords:** fiber optic catalytic probe, plasma probes, atom measurement, oxygen plasma

## Abstract

The presented sensor for neutral oxygen atom measurement in oxygen plasma is a catalytic probe which uses fiber optics and infrared detection system to measure the gray body radiation of the catalyst. The density of neutral atoms can be determined from the temperature curve of the probe, because the catalyst is heated predominantly by the dissipation of energy caused by the heterogeneous surface recombination of neutral atoms. The advantages of this sensor are that it is simple, reliable, easy to use, noninvasive, quantitative and can be used in plasma discharge regions. By using different catalyst materials the sensor can also be applied for detection of neutral atoms in other plasmas. Sensor design, operation, example measurements and new measurement procedure for systematic characterization are presented.

## 1. Introduction

The importance of neutral atoms as a reactive particle in modern sciences is increasing. They are being mainly used in nanoscience [1–4] for synthesis of large quantities of metal oxide nanoparticles, in biomedical science [5–7] for sterilization of delicate biocompatible materials and in surface science [8–10] for changing the surface properties, e.g., activation of polymer materials.

The best way to produce neutral atoms is with electromagnetic discharges. A typical neutral oxygen atom density in low pressure oxygen plasmas is around 10^21^ m^−3^ [11–14]. However, these values can vary for a few orders of magnitude dependent on discharge parameters. For different purposes in terms of treatment of solid materials by reactive oxygen atoms different densities are needed and therefore it is very useful to know the density of atoms in plasmas as precisely as possible. Methods for oxygen atom density measurement include NO titration [15–17], optical absorption spectroscopy [18–20], actinometry [21–23] and catalytic probes [11–14].

NO titration is a pretty reliable chemical method for the determination of O-atom densities, but titration uses toxic NO gas which for safety reasons is usually used in a mixture with Ar. A known amount of NO is leaked in a flowing afterglow region, where NO interacts with O. Excited NO_2_ molecules are formed and then de-excited by light emission. The intensity of this emission is a linear function of NO flow rate and from the slope of this curve the density of O-atoms is determined. The main deficiency of this method is that NO titration can only be used in a flowing afterglow, while in the discharge region NO is destroyed by electron impact. Density in plasma is only estimated with an appropriate model.

Optical absorption spectroscopy techniques are often realized by monitoring the florescence of the laser light absorbed by atoms. Because excitation energies of radiative states suitable for this method are quite high, about 11 eV, at least two photons must be absorbed simultaneously. To achieve this, rather powerful lasers should be used. The deficiencies of these methods are quite expensive equipment and the fact that the method is also only effective in afterglow where atoms are already in the ground state and not in the excited states as they are in the discharge region.

Actinometry is basically optical emission spectroscopy, where a comparison between the intensities of two emission lines, one suitable oxygen emission line and a noble gas (usually argon) emission line, determines O-atom density. In this gas mixture the density of noble gas atoms is known and with the use of intensities ratio we can calculate the density of neutral O-atoms. This method is very popular since it is noninvasive and the equipment is not so expensive, but actinometry is based on an assumption that is usually questionable: it predicts that oxygen atoms are directly excited to a radiative state by electron impact excitation from the ground state.

Among all methods for the estimation of neutral atom density in plasma, catalytic probes are the oldest. The advantages of catalytic probes over other methods are that they are simple, easy to use, quantitative and can be used in plasma discharge regions. They use the fact that a catalyst is heated by the dissipation of energy caused by heterogeneous surface recombination of atoms. From the temperature curve of the catalyst the density of neutral atoms can be calculated. The standard catalytic probe uses a thermocouple to measure the catalyst temperature. The main problem of the standard catalytic probe is EM interference when measurement takes place near RF discharge region. Because the measured values of the thermocouple are in mV range and the wires of a thermocouple act as a receiver the RF interference distorts the electric signal and therefore the measurement is unusable. This is the main reason why a fiber optic catalytic probe (FOCP) has been invented. The EM interference reduction for the case of FOCP probe is proved in the article, where a comparison of fiber optics and standard nickel catalytic probes was made by the inventors of FOCP [24].

## 2. Experimental Section

### 2.1. FOCP Design and Operation

**Figure 1.**
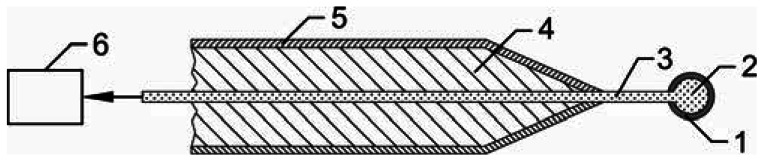
Schematic of the fiber optic catalytic probe (FOCP): 1—catalyst coating; 2—fiber tip; 3—optical fiber; 4—two-component silicone rubber; 5—aluminum housing; 6—optoelectronic detection system.

The schematic of the fiber optic catalytic probe for neutral atom measurement is shown in Figure 1. The FOCP sensor is made from a catalyst foil wrapped around an optical fiber tip, a quartz fiber with diameter of 0.2 mm, aluminum housing filled with two-component silicone rubber (Elastosil M 4370 A/B) and optoelectronic detection device which is optically connected with a remote PC, running an acquisition and processing software.

The housing is made from 9 mm thick surface-oxidized aluminum tube. Oxidation of the Al housing is essential because Al_2_O_3_ is rather inert to atoms such as O, N and H. Therefore practically no surface recombination takes place on the housing and the original concentration of atoms in the chamber is not disturbed much. The tip of the fiber was melted by a brief exposure to Ar arc and, because of the large surface energy of quartz material, a perfect sphere was formed on the fiber tip. In our case the diameter of the sphere was 0.45 mm. The tip is then covered with a catalyst foil.

Depending on the catalyst, the FOCP can be used for measuring different atoms, e.g., nitrogen atoms in N_2_ plasma and hydrogen atoms in H_2_ plasma can be measured with a nitrided iron [25] and a pure gold catalyst, respectively [26]. Here we will present the results for measuring oxygen atoms in O_2_ plasma. For measuring O atoms different catalysts, such as Cu, Nb, Co [27], Ni and Fe [28] can be used. In our case we used a nickel foil of high purity, since the recombination coefficient for O atoms is high and has been determined quite accurately. The value of 0.27 ± 0.04 was found to be independent on temperature in the range from 400 to 900 K [29].

When the probe is placed in oxygen atom-rich atmosphere, extensive surface recombination of the atoms takes place. Because of the energy dissipation on the surface of the catalyst the temperature of the probe rises well above ambient temperature. A standard catalyst probe uses a thermocouple to measure the temperature of the catalyst while FOCP uses an infrared detection system. Radiation in the near IR spectrum, caused by the increased temperature of the catalyst, enters the optical fiber and is transmitted through the fiber to an optoelectronic detection system. This detection system transforms IR radiation to an electric signal and then to optical signal. The photocurrent is converted into digital form and then further digitally processed by a microcontroller. The whole system is then optically linked to a remote PC, where software transforms this digital signal to temperature values [30]. Because of the problems with EM interference the detection system is electromagnetically shielded using solid metal housing.

**Figure 2.**
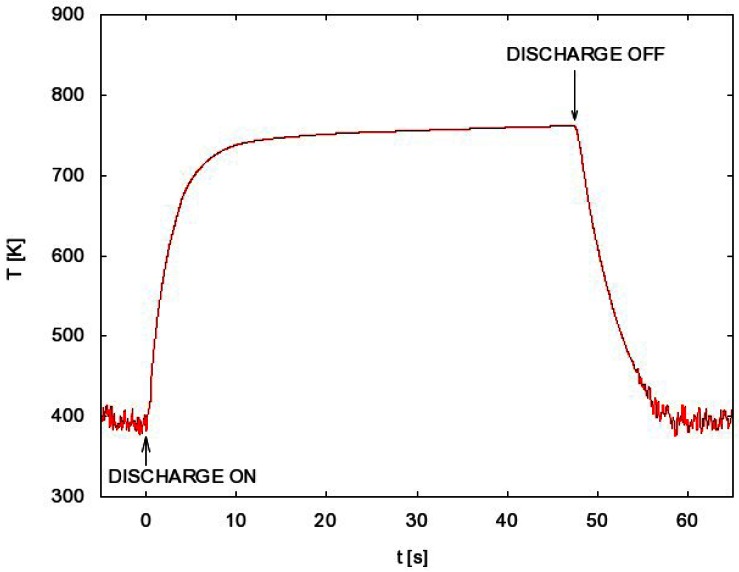
A typical measurement of the probe temperature versus time at a pressure of 55 Pa and a discharge power of 1.2 kW.

As it is shown in Figure 2 the optoelectronic detection system is capable of measuring the catalyst temperature above about 400 K. This detection limit is due to decreasing fiber transmission and detector sensitivity with increasing wavelength of radiation. The lower limit of temperature measurement is therefore the lower limit of FOCP sensitivity.

A newly manufactured FOCP needs to be activated in plasma. Without such activation the probe operation is not reliable and reproducible during subsequent measurements. The term activation stands for removal of any surface impurity and formation of a thin compact oxide film. After activation the temperature and probe signal are calibrated in a furnace. The probe tip is put into a temperature stabilized oven and the measured signal is then fitted with the expression derived from the standard form of Planck spectrum. The detailed calibration is described elsewhere [30].

### 2.2. Atom Density Calculation

When the temperature of the catalytic probe in plasma is measured, we can calculate the atom density in the vicinity of the probe tip.

The catalytic probe is heated at [30]:

(1) $$P_{\text{H}}=j\cdot \gamma \cdot A\cdot \left(\frac{W_{\text{D}}}{2}\right)$$

where *A* is the surface area of the catalyst (8.8 × 10^−7^ m^2^), *γ* is the recombination coefficient (0.27 for O atoms on oxidized nickel surface), *W*_D_ is the dissociation energy (5.12 eV for oxygen [29]) and *j = nv/4* is a particle flux which can be expressed with atom density *n* in the vicinity of the catalytic tip and their average thermal velocity *v* $\left(v=\sqrt{8kT_{g}/\pi m_{a}}=630\text{m}\cdot \text{s}\right)$. Here, *m_a_* is oxygen atom mass, *T_g_* is the gas kinetic temperature in the vicinity of the catalyst and *k* is Boltzmann constant. The kinetic temperature of neutral particles in highly non-equilibrium cold plasmas is usually around room temperature or only slightly higher.

A typical measurement of the FOCP temperature is shown in Figure 2. After igniting the discharge the probe temperature rises until it reaches a constant value. When the constant value is reached the discharge is turned off and the probe temperature starts decreasing.

The cooling rate of the fiber optic catalytic probe is [29]:

(2) $$P_{C}=mc_{\text{p}}\left|\frac{\text{d}T}{\text{d}t}\right|$$

where *m* is the mass of the catalyst (7.6 × 10^−7^ kg), *c*_p_ is its specific heat capacity (444 J·kg^−1^·K^−1^ for nickel) and |d*T*/d*t*| is the absolute value of the time derivative of the probe temperature just after turning off the discharge. The heating and cooling rates are equal at the constant temperature (*P*_H_ = *P*_C_). With rearranging Equations (1) and (2) we obtain an expression for the atom density (*n*):

(3) $$n=\frac{8m\cdot c_{\text{p}}}{v\cdot W_{\text{D}}\cdot \gamma \cdot A}\cdot \left|\frac{\text{d}T}{\text{d}t}\right|$$

Here we see that the atom density can be calculated without the knowledge of the cooling mechanisms whether it is radiation following the Stefan-Boltzman law, thermal conductivity of the fiber, thermal conductivity of surrounding gas or convection.

### 2.3. Plasma Reactor

**Figure 3.**
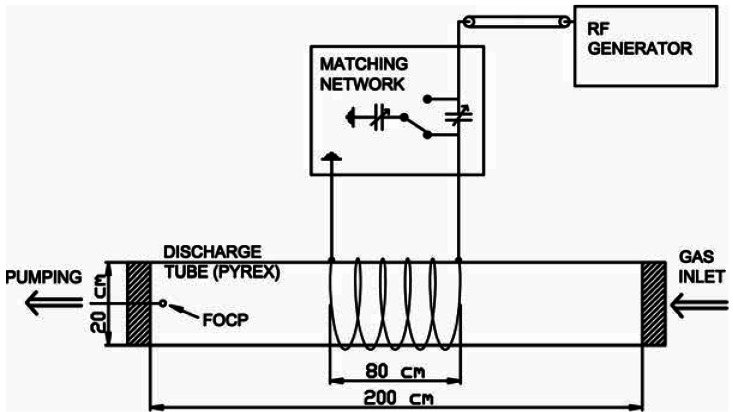
Experiment setup.

All measurements were performed in an inductively coupled RF plasma reactor shown in Figure 3 and described in details in [31,32]. Plasma was created in a large borosilicate glass (Pyrex) discharge tube with a length of 200 cm and a diameter of 20 cm. The discharge tube was pumped by rotary pump with a nominal pumping speed of 80 m^3^·h^−1^. The pressure was measured with an absolute vacuum gauge. The base pressure in the system was about 1 Pa. Oxygen was leaked into the discharge chamber through a precise leak valve.

The excitation coil was placed in the middle of the discharge tube and was connected to the RF generator via coaxial cable and a matching unit. The RF generator is working at a frequency of 27.12 MHz and a maximum power of 8 kW. Matching unit consist of two high-frequency, high voltage, vacuum variable capacitor, whose capacitance can be adjusted with two servo motors.

Experiments were performed in a pressure rage from 10 to 100 Pa and a power range from 0.5 to 4.5 kW. FOCP was placed away from the glow region at the edge of the reactor approximately 1 cm from the aluminum flange.

## 3. Results and Discussion

Now let us present a few examples of the FOCP measurements. When we want to know the atom density at fixed parameters, we need just one measurement, e.g., FOCP temperature *versus* time in Figure 2. From this measurement we extract the time derivative just after turning off the discharge and we calculate the atom density using Equation (3). Usually, however, more than one measurement is needed because the atom density should be determined at different conditions. For each set of parameters one such measurement must be performed.

**Figure 4.**
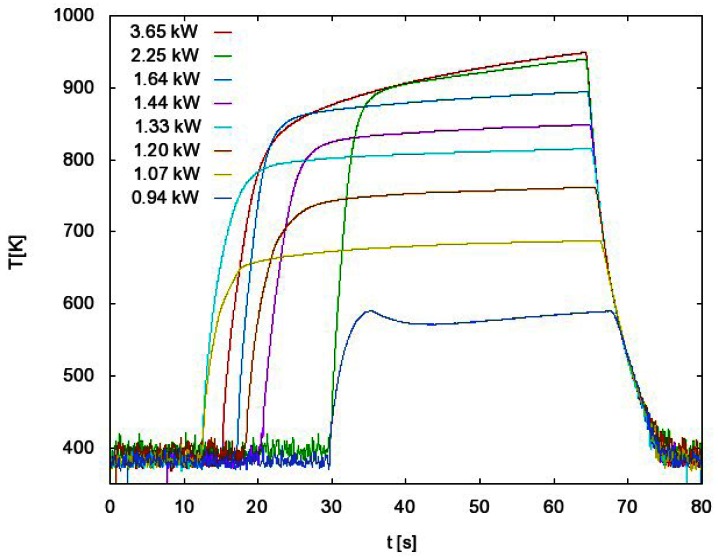
Temperature measurements of the probe *versus* time, the RF generator power is the parameter. Pressure is 55 Pa.

**Figure 5.**
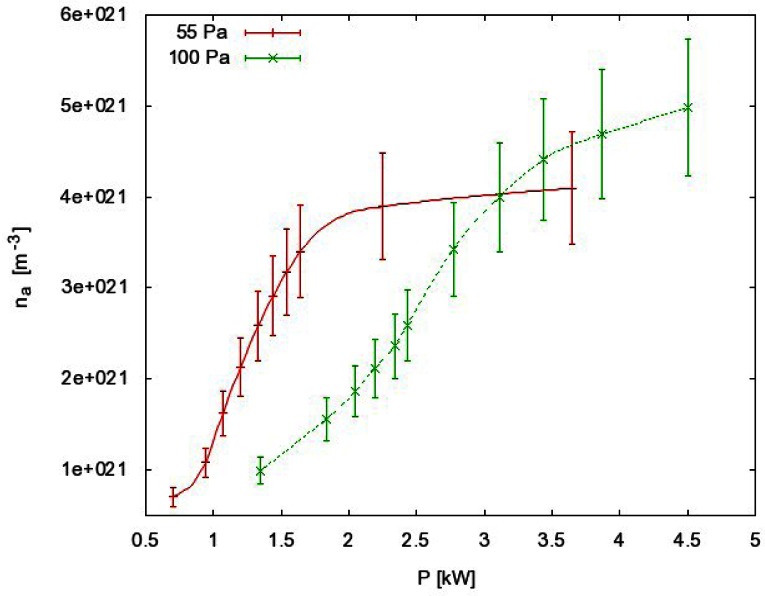
An O-atom density *versus* generator power at 55 Pa (red) and 100 Pa (green).

In the case presented in Figure 4, we measured the atom density at a fixed pressure and we changed the power of the RF generator. For each power we measured the whole temperature evolution: the rise until maximum (constant) temperature is reached and the cooling of the probe after the discharge is turned off. We extracted the time derivative at a maximum temperature just after turning off the discharge and we used Equation (3) to get the behavior of how the atom density changes when generator power is increased (Figure 5, red curve). Similar measurements were performed when FOCP was compared to standard catalytic probe [24] and to NO titration [16].

**Figure 6.**
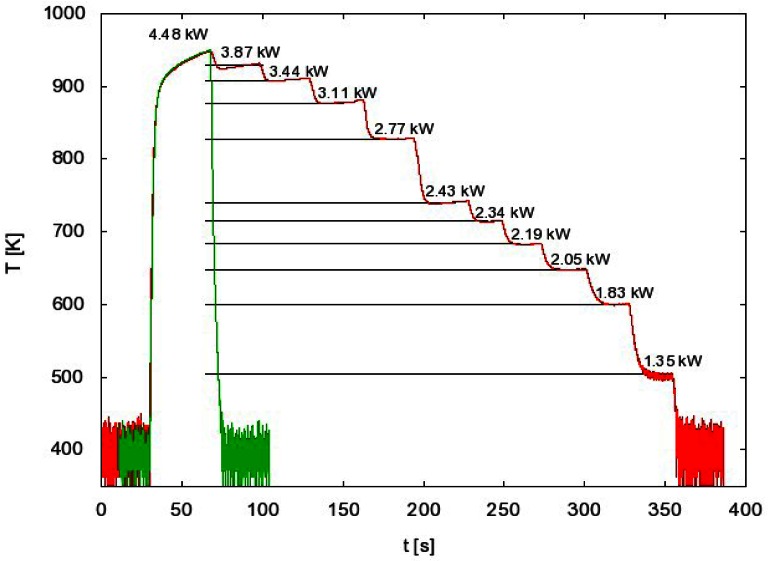
The temperature of FOCP for different generator powers and the whole temperature evolution for maximum generator power at the pressure of 100 Pa.

Such a procedure may be time-consuming when systematic measurements are to be performed at various experimental conditions. In order to simplify the measuring procedure we invented another method of measuring with FOCP that does not involve the whole temperature evolution measurement for each particular experimental condition. This method takes into account the fact that the cooling curve is the same for every generator power as long as the pressure remains constant. This can be seen in Figure 4 where the temperature curves are superimposed. If we use this fact, we can have the same result with only two separate measurements. These two measurements are shown in Figure 6.

The first curve in Figure 6 is the whole temperature evolution at the maximum generator power. From this measurement we get the cooling curve and therefore the time derivative for each temperature.

We start the second measurement with maximum generator power and we wait until the maximum (constant) temperature is reached. Then we suddenly change the power to a lower value and wait until the temperature of FOCP is stabilized. The procedure is then repeated at even lower power values. Using this procedure we only need to extract, from the cooling curve obtained with the first measurement, the time derivative at these stabilized temperatures (Figure 6). With the use of Equation (3), we get the atom density versus generator power (Figure 5, green curve).

Of course, both measurements must be repeated if either pressure or volume flow is changed, since the cooling curve is different for each pressure. Here it is worth mentioning that the density values in Figure 5 have been calculated taking into account an assumption that only recombination of atoms contributes to the probe heating, while the contributions of other particles are negligible. This fact is almost trivial when the measurement takes place in an afterglow since only neutral atoms survive many surface collisions on the way from the discharge to afterglow. The probability for heterogeneous surface recombination of neutral atoms is often below 10^−3^ on glass materials. Also neutral atoms are stable in vacuum because a three body collision is required for their recombination, and at low pressure these collisions are very improbable.

However in plasma, in the discharge region, the situation is different since there are also ions and excited atoms which could contribute to the heating of the probe. Not just the recombination of atoms dissipates the energy on the catalyst surface but also relaxation of excited atoms and neutralization of ions as well as weak bombardment by ions since the probe is kept at floating potential. Therefore in the Equation (1) at least three more contributions would have to be added. Luckily enough these contributions are so small that we can neglect them. The reason is that the density of charged particles and excited atoms is typically many orders of magnitude lower than the density of neutral atoms. Using a double Langmuir probe, measurements of ion density in similar plasmas have been already performed and it was usually around 10^16^ m^−3^ [33]. The density of excited O-atoms in the first excited state (^1^D_2_) was measured by vacuum ultraviolet laser absorption spectroscopy. In a microwave discharge created in noble gases with small concentration of oxygen the density of excited O-atoms was around 10^18^ m^−3^ [34]. On the other hand, typical neutral atom densities are of the order of 10^21^ m^−3^ [11–14] and may exceed the value of 1 × 10^22^ m^−3^ so the simplification neglecting other heating mechanism is justified in the case when plasma is created by an electrodeless discharge created in glass discharge chambers.

Measurement errors (error bars in Figure 5) are mainly due to systematical errors such as inaccuracy of recombination coefficient, surface area of the catalyst and mass of the catalyst. They add up to about 20%. Statistical errors however are almost negligible, since measurements show excellent repeatability. Repeatability of results obtained with FOCP is better than 1% [24].

So far around 40 FOCPs were manufactured and were used in quite a few experiments where characterization of plasma was needed. They were also compared with other methods for measuring the density of neutral oxygen atoms. The accuracy of FOCP was determined by comparison of the results obtained by FOCP and standard catalytic probes. The agreement was within ±20% [30].

Our probes also showed excellent performance in experiments where they were compared with another standard method for determination of the neutral oxygen atom density—chemical titration using NO gas [16]. Furthermore, comparison of our probes with another method for quantitative determination of the atom density in the plasma afterglow, TALIF (Two-Photon Absorption Laser Induced Fluorescence), showed extremely good agreement [35]. The absolute accuracy of the probes was estimated to be similar to other methods—about 30%.

## 4. Conclusions

A sensor for neutral oxygen atom measurement in oxygen plasma was presented. The design and operation of the sensor and atom density calculation was described in detail. A comparison with other techniques for determining oxygen atom densities shows that the sensor described in this paper has a few advantages: it is simple, easy to use, quantitative and can be used in plasma discharge regions. The latter advantage was explained to detail and justified in this paper. A new measurement procedure was invented in order to reduce the number of separate measurements, and it is particularly useful when systematic plasma characterization is to be performed. With this procedure it is possible to obtain the results in less than half the time of the normal measurements. Even though this paper is focused on nickel FOCP used for measuring neutral oxygen atom density, with the use of a different catalyst material FOCP can also be useful for detection of other neutral atoms in plasmas created in other gases.
